# Investigation of Cell Adhesion and Cell Viability of the Endothelial and Fibroblast Cells on Electrospun PCL, PLGA and Coaxial Scaffolds for Production of Tissue Engineered Blood Vessel

**DOI:** 10.3390/jfb13040282

**Published:** 2022-12-08

**Authors:** Morteza Bazgir, Morvarid Saeinasab, Wei Zhang, Ximu Zhang, Ka Min Tsui, Abolfazl Maasoumi Sarvestani, Subhaan Nawaz, Phil Coates, Mansour Youseffi, Jacobo Elies, Farshid Sefat

**Affiliations:** 1 Department of Biomedical and Electronics Engineering, School of Engineering, University of Bradford, Bradford BD7 1DP, UK; 2 Department of Biology, Faculty of Science, Ferdowsi University of Mashhad, Mashhad 9177948974, Iran; 3 State Key Laboratory of Polymer Materials Engineering, Polymer Research Institute, Sichuan University, Chengdu 610065, China; 4 Advanced Polymer Materials Research Centre, Sichuan University, Shishi 362700, China; 5 Chongqing Key Laboratory of Oral Disease and Biomedical Sciences and Chongqing Municipal Key Laboratory of Oral Biomedical Engineering of Higher Education, Stomatological Hospital of Chongqing Medical University, Chongqing 401174, China; 6 Interdisciplinary Research Centre in Polymer Science and Technology (Polymer IRC), University of Bradford, Bradford BD7 1DP, UK; 7 School of Pharmacy and Medical Sciences, Faculty of Life Sciences, University of Bradford, Bradford BD7 1DP, UK

**Keywords:** tissue engineering, electrospinning, polycaprolactone (PCL), poly (lactic-co-glycolic acid) (PLGA), cell viability, cell proliferation, human umbilical vein cells (HUVEC), human vascular fibroblast cells (HVF)

## Abstract

Endothelialization of artificial scaffolds is considered an effective strategy for increasing the efficiency of vascular transplantation. This study aimed to compare the biophysical/biocompatible properties of three different biodegradable fibrous scaffolds: Poly (ɛ-caprolactone) (PCL) alone, Poly Lactic-co-Glycolic Acid (PLGA) alone (both processed using Spraybase^®^ electrospinning machine), and Coaxial scaffold where the fiber core and sheath was made of PCL and PLGA, respectively. Scaffold structural morphology was assessed by scanning electron microscope and tensile testing was used to investigate the scaffold tension resistance over time. Biocompatibility studies were carried out with human umbilical vein endothelial cells (HUVEC) and human vascular fibroblasts (HVF) for which cell viability (and cell proliferation over a 4-day period) and cell adhesion to the scaffolds were assessed by cytotoxicity assays and confocal microscopy, respectively. Our results showed that all biodegradable polymeric scaffolds are a reliable host to adhere and promote proliferation in HUVEC and HVF cells. In particular, PLGA membranes performed much better adhesion and enhanced cell proliferation compared to control in the absence of polymers. In addition, we demonstrate here that these biodegradable membranes present improved mechanical properties to construct potential tissue-engineered vascular graft.

## 1. Introduction

The ability to fabricate synthetic, functional, and successful human body parts has always been a goal of the human exploration of science. The evolution of vascular surgery began in 1896 when Jaboulay and Briau successfully performed arterial transplantation of the carotid artery [1]. It was surprising, given that they already held the belief that sutures placed in the vessel would result in its premature thrombosis, yet anastomoses were imperfect and caused thrombosis. A year later in 1897, a successful end-to-end arterial anastomosis of the femoral artery was performed by John Murphy. This landmark procedure followed significant experimental work with vascular anastomosis in canine and bovine subjects, and it paved the way for the subsequent advances in the next century [2]. Since the late 19th century, more sophisticated techniques have been discovered and developed, and to this day, autografts are routinely used in surgery as a gold standard method for replacing damaged blood vessels. However, the use and availability of autografts are limited, especially for arteries. This led to researchers and scientists to investigate and develop an alternative method for replacing arterial vessels rather than using the conventional method.

The artificial blood vessel was successfully developed and began clinical application in the 1950s. Voorhees discovered the first artificial porous scaffold, producing a tubular graft by folding and stitching a Vinyon “N” cloth to the desired sizes. To validate the effectiveness of the scaffold, an in vivo study was carried out in dogs, with a single tube placed in the abdominal aorta. The preliminary results were promising, which encouraged more scientists to discover and develop alternative successful artificial blood vessel prosthesis [3]. More complex methods have been implemented since then, and many different types of artificial blood vessel prosthesis have been developed.

Currently, most synthetic blood vessels in clinical use are made from synthetic non-degradable materials; this is mainly due to the ease and flexibility of modifying mechanical properties. Most of these synthetic materials were introduced by the end of the 1970s as a solution for aortic and lower extremity bypass, mainly composed by PTFE (Teflon) [4,5,6] and Dacron [7]. These non-degradable synthetic grafts proved to be useful as an alternative method to traditional autologous vessels or bypass grafting procedure. However, it has not been considered a long-term solution due to the relatively poor patency rate of these synthetic materials over a long period of implementation. This is the standard limitation of these vascular grafts that have driven investigation of different strategies.

Using a biodegradable polymeric scaffold on which cell layers can grow is an alternative tissue engineering technique for the development of a functional long-term vascular graft [8]. The principle of using these types of degradable structure is that the scaffold is replaced and reconstructed by extracellular matrix (ECM) secreted by the cells. Many alternative approaches to blood vessel grafts have been developed, combining the concepts of bioresorbable and biocompatible polymers, those that are not toxic to the body on implementation, and can exhibit controlled degradation resorption in the body. 

In the last two decades, approaches taken to create the best tissue-engineered vascular grafts were mainly focused on the surface coating and chemical modification of synthetic materials [9,10,11,12], biodegradable scaffolds, and biopolymer [13,14]. Many processing techniques have been investigated to produce synthetic porous vascular graft scaffold, and electrospinning has emerged as a new scaffold fabrication method. This fabrication method can produce interconnected meshes with fibers ranging from few nanometers to several micrometers in diameter that mimics the extracellular matrix of native tissue [15,16]. It has been noted that most of the polymeric scaffolds produced by electrospinning procedure had outstanding structural integrity when compared to other conventional fabrication methods. Many synthetic aliphatic polyesters such as PCL [17], PLLA [18], PGA [19], PLGA [20], and PLA [21], which have been conventionally and commonly used for producing a variety of tissues, have shown excellent processability in forming nanofibrous mesh through electrospinning. More recently, we compared the biomechanical properties of PCL, PLGA, and combination of these two polymers in either bilayer or coaxial conformation, demonstrating that coaxial conformation present superior mechanical properties for the design of artificial vascular grafts [22,23]. In this study, we show evidence of the superior biocompatibility properties of the combination of PCL and PLGA in a coaxial conformation by investigating the compliance regarding cell adhesion and proliferation.

For tissues and organs to function appropriately, scaffolds must be designed to promote direct cell transfer to three-dimensional structure and support tissue reconstruction [24,25,26]. The tissue engineering approach provides an attractive method for transplanting cell vessels, the primary strategy being to create tubular scaffolds by attaching autologous cells to natural, synthetic, or hybrid porous scaffolds under suitable in vitro conditions [27,28].

The surface of the scaffold is the central part that interacts with surrounding cells and tissues. Since most cells used in tissue engineering depend on attachment, it can be interpreted that the material and the architecture of the scaffold should promote cell attachment. Therefore, a membrane with large and accessible pores is desired [29]. In tissue engineering, membranes that have been produced by bioresorbable polymers are more advantageous than grafts that are made of the non-degradable polymer because the biodegradable scaffold implant with sufficient mechanical properties can be used as a solution to replace the damaged organs by dissolution slowly over some time, leaving behind no toxic synthetic materials that can lead to further reaction or inflammation with a foreign body [30,31].

The importance of macro-porosity in electrospun fibers is highlighted by the pores that allow cell penetration into the electrospun mat, considering the fibrous structure’s density [32,33]. Usually, cells can stick to the surface without penetrating the surface pores of the scaffolds [34]. The interaction between these dimensions and porosity is a fundamental element of tissue engineering related to cell type and the target tissue [35]. Modern prosthetic small-diameter coronary grafts are often blocked and cause complete failure due to early thrombosis (blood clot) and late internal enlargement [36]. The reason for choosing PLGA is that compared to commonly used synthetic polymers (such as PLLA and PCL/PU), it performs better in cell survival, allows for pseudo vascularization in vitro, and cell proliferation as well as decrease in thrombosis and hyperplasia [37,38]. Scaffolds that have been fabricated from these types of biodegradable polymers are preferred due to the endothelial layer can be formed easily on the surface of the synthetic grafts, and this kind of layer on the scaffold surface can help to prevent the formation of thrombosis and hyperplasia [39]. As the method used to fabricate the polymeric scaffold is electrospinning, all areas of the electrospun scaffold must be covered by endothelial cells, since many studies have demonstrated how easily thrombosis can occur when there was an absence of a complete endothelium [40,41,42,43]. Although biodegradability and biocompatibility are well-studied properties of PLGA polymer, the fragility and significant degradation rate associated with this synthetic polymer restrict its use in tissue regeneration purposes [44]. PCL is a known flexible biodegradable polyester that can overcome the vulnerability and low elongation characteristics of PLGA; it is more hydrophilic than PLGA, and encourages more cell adhesion and proliferation [45].

Having an adequate endothelial cell layer will reduce the risk of tissue-engineered blood vessel failure due to thrombosis. Since the endothelial cell surface is rich in heparin-like glycosaminoglycans, and the microvascular endothelium is the leading site of active thrombin inactivation in the body, the combination with these glycosaminoglycans also provides a rich source of the antithrombin function [46,47,48]. Vascular fibroblasts and smooth muscle cells are critical for extracellular matrix construction, vasoconstriction, and vasodilatation, and for these reasons, they enhance functionality of tissue-engineered blood vessels in vivo [49,50,51]. Hence, developing a stable synthetic blood vessel membrane requires a deep understanding of how vascular cells interact in vitro. The purpose of this work is to study the biological response of biodegradable scaffolds synthesized with specific cells, namely human vascular fibroblasts (HVF) and human umbilical vein endothelial cells (HUVEC).

## 2. Experimental

This study focuses on the validation of fabricated biodegradable grafts for tissue-engineered vascular graft (TEVG) purposes with a strong emphasis on cell viability, adhesion, and proliferation of vascular cells into electrospun polymers. The characterization processes consisted of morphological, durability and biological assessments. The materials and methods used for validation are as follows.

### 2.1. Scaffold Materials

PURASORB poly (lactic-co-glycolic acid, PLGA) 82:18 obtained from Corbion Netherlands and Poly (ɛ-caprolactone, PCL) with an average molecular weight wt. Mn 80,000 g/mol and density of 1.145 g/mL at room temperature, purchased from Sigma-Aldrich. Chloroform (CF) supplied by Fisher Scientific, UK, without prior purification were used as solvents.

### 2.2. Solution Preparation and Electrospinning Process

The solution was prepared by dissolving 1.7 g of PCL pellets in 8.3 g of chloroform and 1.4 g of PLGA in 8.6 g of chloroform. The solutions were placed on the magnetic stirrer in a sealed sterile glass container for a minimum of 24 h; then when the homogenized viscous solution was observed, the glass vials were placed in an ultrasonic bath for 3 h minimum to eliminate air bubbles that produced while mixing the product. Before the electrospinning procedure, polymeric solutions were drawn in the sterile 5 mL NORM-JECT syringe and then mounted to the syringe pump. An 18G needle was used for electrospinning the PCL and PLGA solutions, and for coaxial scaffolds, a coaxial needle was used, which the PCL solution was pumped through the inner needle (core) and the PLGA solution for the outer needle (shell). The size of the tip of the coaxial needle is VITA 20, which is noticeably smaller than the 18G needle. All three scaffolds were electrospun for 60 min, with a flow rate of 1 mL/h. The voltage was increased when it was necessary for obtaining a Tylor cone at the tip of the needle. Table 1 below provides a summary of the parameters recorded during the electrospinning procedure.

### 2.3. Scaffold Morphology

The morphology of the electrospun scaffolds was observed by using scanning electron microscopy (SEM) with a 15kV accelerating voltage, Hitachi TM3000, Japan. The images were taken at 2500X magnification. The average fiber diameter (µm), average pore size (µm)^2^, and scaffold surface porosity percentage were determined using SEM-assisted image analysis software (ImageJ software). At least 20 fibers and 20 pores are analyzed for each sample image, and the average value of each sample was determined.

### 2.4. Scaffold Tensile Testing Process

The mechanical properties of the electrospun scaffolds were measured with a uniaxial testing machine (MACH-1 uniaxial mechanical tester). The samples were cut in a rectangular shape with dimensions of 50 × 10 mm using surgical scissors. The thickness of each sample was measured via both digital micrometer and digital caliper. The tensile test was conducted using a single-axis 10 kg load cell under the velocity of 0.5 mm/s at room temperature condition. At least four samples were tested for each type of electrospun mesh.

### 2.5. Scaffold Sterilization Process for Cell Culture Work

Before proceeding with cell culture experimental work, excess organic solvents was first removed using a sterile vacuum chamber at room temperature for a minimum of 24 h, then scaffolds were transferred into a vacuum oven for additional 24 h at 37 °C. Scaffolds were then sterilized by direct exposure to ultraviolet (UV) light (8 h on each side) and then placed in a sterile vacuum plastic bag until the day of cell culture studies. The bags containing the biodegradable polymeric scaffolds were sterilized with 70% ethanol to avoid cross-contamination between engineering and cell culture lab. Additionally, the scaffolds also were soaked with 70% ethanol for 10 min, then rinsed twice with phosphate-buffered saline (PBS) solution to remove any ethanol residues.

### 2.6. Cell Culture

Human umbilical vein endothelial cells (HUVEC, Cellworks ZHC-2102) and human vascular fibroblast cells (HVF, Human aortic adventitial fibroblasts, PromoCell, Cat No C-12380) were cultured in endothelial cell growth medium MV (PromoCell C-22020) and fibroblast growth medium 2 (PromoCell C-23020) supplemented with 10% FBS, respectively. Cells were maintained in a cell culture incubator at 37 °C and 5% CO_2_ (humidity saturated) to ensure the pH was kept within the physiological range. Media was replaced every 2–3 days and cells were trypsinized when cell density in flask reached 70%.

### 2.7. Cell Viability Assay (MTT)

The MTT assay was performed to investigate the viability of cells on the synthetic PCL, PLGA, and Coaxial scaffolds. Fabricated scaffolds were cut into 0.32 cm^2^ disc pieces and placed in 96-well plates. Before cells were seeded, scaffolds were soaked in the corresponding cell culture medium for at least 24 h to improve their hydrophilicity. HUVEC and HVF cells were seeded at a density of 10,000 cells/well on top of 1 (1:1), 2 (1:2), or 3 (1:3) 0.32 cm^2^ polymeric scaffold discs. MTT assays were conducted after 72 h of culture using CellTiter 96^®^ AQueous Non-Radioactive Assay (Promega; Cat. No. G5421). After incubating the plate at 37 °C for 2 h, absorbance readings at 570 nm were obtained using a plate reader spectrophotometer.

### 2.8. Proliferation Assay

Cell proliferation assays were conducted in 24-well plates (Nunc^TM^) where HUVEC and HVF were seeded on top of sterile disc pieces (1.9 cm^2^) of polymeric scaffolds. Trypsin was used to detach cells from the scaffolds (or plastic in the case of control wells). After that, scaffolds were rinsed (and removed from the wells) with complete media. The same complete media was used to stop the action of trypsin in each well. Then, cell suspensions were centrifuged (300× *g* for 5 min) and cell pellet was resuspended in PBS prior to manual cell counting. Trypan blue was used to distinguish live and dead cells. Manual cell counting was conducted after 24, 48 and 72 h, using a hemocytometer chamber under the microscope.

### 2.9. Fluorescence Microscopy (Nuclear Staining with DAPI and Immunofluorescence)

Similarly, as described above, HUVEC and HVF cells were seeded onto the three fabricated scaffolds and maintained in the cell culture incubator for 48 h. Before staining, cell plates were rinsed twice in PBS to remove the excess of phenol red. 4′,6-diamidino-2-phenylindole (DAPI) (Sigma) and CellMask^TM^ (Molecular Probes, Invitrogen) were used to visualize the nuclei and cell membranes, respectively, following manufacturer instructions. After staining, cells were fixed with 4% formaldehyde. Images of the membrane surface were captured using confocal microscopy.

### 2.10. Statistical Analysis

Data were expressed as mean ± standard deviations (SD). Initial tests were performed to determine the natural distribution of the data and the appropriateness of parametric or non-parametric tests. Average and standard deviations of fiber diameter and pore size were determined using Microsoft Excel. Analysis of variance (one way ANOVA test) was used to determine *p*-values to infer any difference in the cell viability/proliferation performance of the scaffolds and compared to the control (absence of scaffold). The differences were considered statistically significant at *p* < 0.05.

## 3. Results and Discussion

Some of the factors which can influence attachment, migration and proliferation of cardiovascular cells are associated with the topography and chemical composition of the scaffold. In this work, we have fabricated three distinct micro-fibrous electrospun scaffolds composed of PCL 17 wt% PCL, 14 wt% PLGA, and coaxial (core-17 wt% PCL and shell-14 wt% PLGA), characterized their fiber diameter, pore size, porosity, and mechanical properties; and then evaluate their biocompatibility and adhesion properties in human vascular endothelial cells (HUVEC) and fibroblasts (HVF).

**Figure 1:**
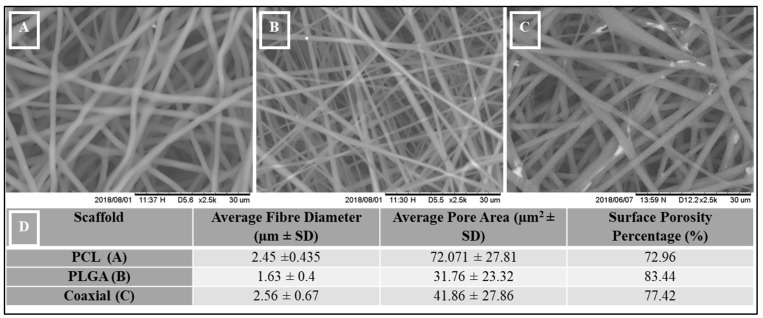
Scanning Electron Microscope (SEM) images, (**A**) 17% PCL, (**B**) 14% PLGA and (**C**) Coaxial (17% PCL / 14% PLGA), (**D**) Measurements of Morphological Structural Parameters of PCL, PLGA, and Coaxial.

Figure 1 shows the morphology of PCL (A), PLGA (B), and coaxial scaffold (C) captured by scanning electron microscopy (SEM) with a magnification at 2500X. The three scaffolds show similar bead-free, porous mesh topography, with relatively uniform ultrafine fibers. Randomly oriented fibers describe the interconnected structure, with an average fiber diameter of 2.45 µm ± 0.435 SD, 1.63 µm ± 0.4 SD, and 2.56 µm ± 0.67 SD corresponding to the PCL, PLGA, and coaxial scaffolds, respectively (Figure 1D).

Interestingly, PCL scaffold showed the largest average pore size (72.07 µm^2^ ± 27.81 SD) and compared to the other two membranes, PCL showed the lowest surface porosity percentage (Figure 1D).

In 1996, Recum initially investigated surface roughness, porosity, and texture as modifiers of cellular adhesion [52] and eight years later, the significance of having a massive pore area greater than 250µm have been illustrated by Druecke [53] and other researchers [54,55].

**Figure 2:**
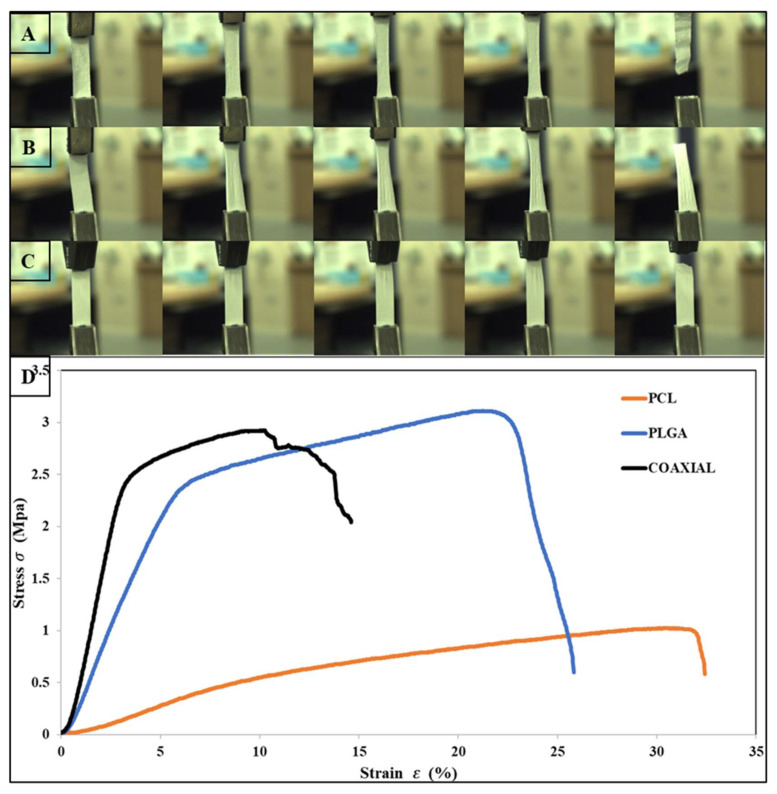
Photographs of flat sheet scaffolds during tensile testing, (**A**) PCL, (**B**) PLGA, (**C**) Coaxial, and (**D**) show the stress–strain curves obtained for each sample.

The tensile properties for electrospun 17 wt% PCL, 14 wt% PLGA, and coaxial (core = 17 wt% PCL and sheath = 14 wt% PLGA) are shown in Table 2. The stress–strain curves of the fibrous membranes are also presented graphically in Figure 2. The tensile strength of PCL, PLGA, and the coaxial electrospun sheet was 1.02 MPa ± 0.2 SD, 3.1 MPa ± 0.57 SD, and 2.89 MPa ± 0.97 SD, respectively. Previous studies have shown that coaxial electrospun scaffolds have many advantages over other electrospinning process counterparts, particularly the ability to produce fiber core materials with desired mechanical properties and the sheath made of biocompatible materials, which interacts better with host tissues [56,57,58]. However, in this study, the coaxial specimen did not show better mechanical characteristics, especially compared to the PLGA membrane. Surprisingly, the PLGA membrane had better and higher tensile strength and was more elastic than the coaxial membrane with elongation at break 25.94 % ± 3.56 SD and 14.61 % ± 4.0 SD, respectively. Two leading phenomena can cause such improvement to the mechanical properties of the electrospun PLGA membrane: (i) Polymeric solution concentration—either due to the mechanical properties of the PLGA polymer or because the fabricated electrospun membrane was produced from a high polymeric solution concentration (14 wt%); and (ii) Increased lactide/glycolide ratio, as a higher ratio of lactide (LA) to glycolide is associated with improved mechanical properties of the scaffold [59,60].

In this study, we sought to compare the biocompatibility properties of three distinctive polymeric fibrous scaffolds for the development of vascular stents, and for that reason we chose to test the biocompatibility in two human vascular cell types: endothelial cells (HUVEC) and vascular fibroblasts (HVF). In addition, cell culture can quickly provide information about how specific cell types respond to biomaterials and reduces the needs for animal testing at most stages of biomaterial development [61].

**Figure 3:**
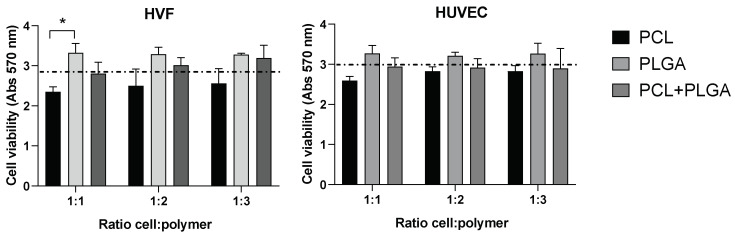
Cell viability (MTT) of HVF (left graph) and HUVEC (right graph) in PCL, PLGA, and coaxial (PCL+PLGA) scaffolds. Dash line represents the cell viability in the absence of scaffold (control condition). Each bar represents mean (and SD) of HVF (left) and HUVEC (right) cell viability in each distinctive scaffold as indicated in the graphs. Note that cell viability in the presence of scaffolds is not significantly different from the control (one way ANOVA: * *p* < 0.05; *n* = 3 independent experiments).

We investigated the potential cytotoxicity of the polymeric scaffolds by culturing both cell types at three different cell densities (1:1, 1:2, and 1:3) in the presence of the distinctive polymeric PCL, PLGA and coaxial electrospun scaffolds. Our results did not show significant differences between the cell viability in the presence of the polymeric scaffolds compared to the control (absence of scaffold) (Figure 3). In addition, differences in cell density did not affect the cell viability in any of the scaffolds, indicating that cell viability (for both HVF and HUVEC) is not affected by the amount of polymer present in the experimental conditions.

Although the differences in the variance of the average cell viability among the three polymeric scaffolds is not statistically significant, there is a trend across the three cellular densities tested in HVF and HUVEC showing a higher cell viability average for the PLGA polymer compared to PCL and control condition. Importantly, PLGA polymer showed higher cell viability than PCL polymer when HVF were seeded at a 1:1 cell density, however, this increase in cell viability was not statistically significant in the case of HUVEC or when HVF were seeded at higher density.

**Figure 4:**
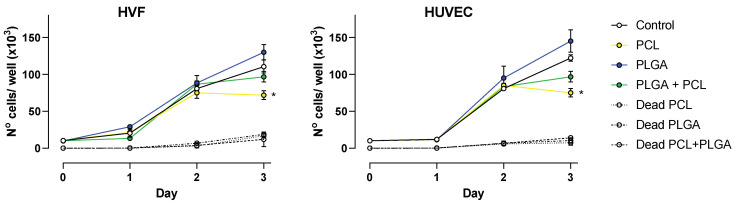
Cell proliferation over a period of 3 days. Plots show average number of cells ± SD at 24, 48 and 72 h for HVF (left panel) and HUVEC (right panel). Dead cell counting is represented as indicated per each polymer (dash line traces), * *p* < 0.05.

For fabricated polymeric scaffolds to be biocompatible, they must promote cell adhesion and proliferation. A cell proliferation assay was conducted with HUVEC and HVF cells to unveil the influence of PCL, PLGA and coaxial porous membranes on supporting cell proliferation. The results obtained from cell counting are shown in Figure 4.

At 24 and 48 h, both vascular cell types showed a similar growth rate as the control condition (absence of polymers); however, at 72 h the presence of polymeric scaffolds had a significant impact in the cell proliferation.

Our results indicate that in the presence of PLGA scaffolds, HVF and HUVEC cell proliferation occur in a similar fashion as control conditions, and although on average the number of cells was higher in the presence of PLGA scaffolds, the difference was not statistically significant compared to control at 72 h. On the contrary, PCL scaffolds decrease cell proliferation in both HVF and HUVEC, and this becomes significant at 72 h. This finding is also similar for the coaxial scaffold due to the membrane fiber consisting of both PCL and PLGA, but under our experimental conditions the decrease in cell proliferation is not significantly different compared to the control (Figure 4).

In addition, we determine the number of dead cells present in each sample. The number of dead cells at 24 and 48 h was below the detection range of the hemacytometer technique. At 72 h, the number of dead cells in the presence of polymeric scaffolds was on average 8.7 × 10^3^ cells/mL, and values were not significantly different to the number of dead cells in the control wells, suggesting that the presence of polymer does not influence cell death. This agrees with the results obtained by MTT reported above.

**Figure 5:**
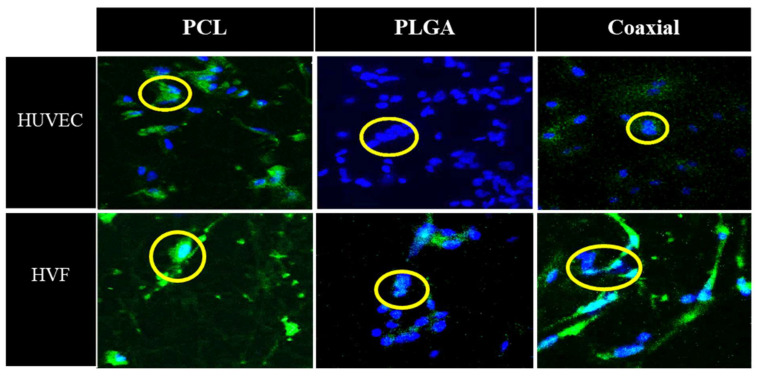
Representative confocal microscopy images of cultured human umbilical vein cells (HUVEC) and human vascular fibroblast (HVF) seeded in polymeric PCL, PLGA, and coaxial scaffolds. Nuclei were stained with DAPI (blue) and cell membranes are labelled with CellMask (green).

Figure 5 represents confocal microscopy images of 5-day cultured human umbilical vein cells (HUVEC) and human fibroblast cells seeded on fibrous polymeric PCL, PLGA and coaxial scaffolds. In the case of HUVEC cells, all samples were confirmed to have adhesion capacity, which demonstrates the biocompatibility of such membranes. The PCL scaffold showed a smaller amount of fibroblast cells than PLGA and coaxial membranes, which approximately about 14 nuclei could be counted for each of the membranes.

In summary, our results obtained from cell proliferation assays and confocal microscopy have shown favorability for the eventual use of these polymers (in combination or alone), with certain adjustments and optimization of conditions, to construct semi-synthetic blood vessels.

## 4. Conclusions

In this study, three different biodegradable electrospun fibrous membrane types were successfully fabricated from 17 wt% PCL and 14 wt% PLGA solutions. Cell adhesion and proliferation of HUVEC and HVF to the synthetic electrospun fibrous membranes were found to be rather complicated and challenging without the addition of agents/ peptides which promote cell attachment (ex. gelatin, collagen, VEGF). The results obtained from cell viability assay and confocal imaging demonstrated that these synthetic biodegradable membranes are suitable to support adhesion and proliferation of vascular cells. Apart from PCL scaffolds, which showed a significant decrease in HVF and HUVEC proliferation at day 3, our results demonstrate that the polymeric scaffolds are non-toxic and have the potential to be used as primary biomaterials for the development of vascular stents. In contrast, other scaffolds such as those fabricated with coaxial needle encouraged the cells to proliferate and differentiate like the PLGA membrane with more promising results. PLGA membrane also showed adequate mechanical properties, and these findings further confirm that PLGA has a higher potential for tissue engineering applications, particularly for blood vessel applications.

Overall, the physiochemical properties, mechanical characteristics, biocompatibility and biodegradability of the PLGA and coaxial porous membranes showed to be ideal for the design of semi-synthetic blood vessels. Our future work will be focused on optimising the biocompatibility of the synthesized polymers by adding naturally occurring biomaterials (ex. collagen, elastin and hyaluronic acid) in combination with the design of multi-layered tubular scaffolds to mimic blood vessels. Further research to improve endothelialization and antithrombotic properties via modification of bioactive factors will be needed.

## Figures and Tables

**Table 1:** Recorded parameters during the electrospinning process.

Electrospinning Sample	Voltage (KV)	Needle Type	Distance from Tip of the Needle to the Collector (mm)	Type of Collector	Flow Rate (mL/h)	T (°C)	Humidity (%)	Time (min)	Solution Dispensed (mL)
17 wt% PCL	10.96	18G	90	Flat	1	26.1	54	60	0.998
14 wt% PLGA	9.29	18G	90	Flat	1	28.2	49	60	1.06
Coaxial PCL(Core)/ PLGA(Shell)	11.31	Coaxial needle	90	Flat	Pump1: 0.5 Pump2: 0.5	22.4	38	60	Pump1:0.495 Pump2:0.511

**Table 2:** Mechanical Properties of each PCL, PLGA, and coaxial electrospun scaffolds.

Sample Name	Length (mm)	Width (mm)	Thickness (mm)	Area (mm)^2^	Tensile Strength (MPa ± SD)	Elongation at Break (% ± SD)	Young Modulus (MPa ± SD)
PCL	46.3	10.7	0.09	0.963	1.02 ± 0.2	32.43 ± 4.37	7.75 ± 5.1
PLGA	47.5	10.3	0.12	1.236	3.1 ± 0.57	25.94 ± 3.56	13.51 ± 2.35
Coaxial	49.41	9	0.09	0.81	2.89 ± 0.97	14.61 ± 4.0	9.18 ± 1.76

## Data Availability

Not applicable.
